# Time-Resolved Spectroscopy of Fluorescence Quenching in Optical Fibre-Based pH Sensors

**DOI:** 10.3390/s20216115

**Published:** 2020-10-27

**Authors:** Katjana Ehrlich, Tushar R. Choudhary, Muhammed Ucuncu, Alicia Megia-Fernandez, Kerrianne Harrington, Harry A. C. Wood, Fei Yu, Debaditya Choudhury, Kev Dhaliwal, Mark Bradley, Michael G. Tanner

**Affiliations:** 1 Scottish Universities Physics Alliance (SUPA), Institute of Photonics and Quantum Sciences, Heriot-Watt University, Edinburgh EH14 4AS, UK; d.choudhury.hw@gmail.com (D.C.); M.Tanner@hw.ac.uk (M.G.T.); 2 EPSRC Proteus IRC Hub, Centre for Inflammation Research, Queen’s Medical Research Institute, University of Edinburgh, 47 Little France Crescent, Edinburgh EH16 4TJ, UK; Tushar.choudhary@roslin.ed.ac.uk (T.R.C.); Kev.Dhaliwal@ed.ac.uk (K.D.); Mark.Bradley@ed.ac.uk (M.B.); 3 Institute of Biological Chemistry, Biophysics and Bioengineering, School of Engineering & Physical Sciences, Heriot-Watt University, Edinburgh EH14 4AS, UK; 4 School of Chemistry, EaStChem, University of Edinburgh, Joseph Black Building, West Mains Road, Edinburgh EH9 3FF, UK; muhammed.ucuncu@ikc.edu.tr (M.U.); v1amegia@exseed.ed.ac.uk (A.M.-F.); 5 Centre for Photonics and Photonic Materials, University of Bath, Claverton Down, Bath BA27AY, UK; kh1c19@soton.ac.uk (K.H.); H.Wood@bath.ac.uk (H.A.C.W.); yufei@siom.ac.cn (F.Y.)

**Keywords:** fluorescence spectroscopy, time-resolved spectroscopy, optical fibre sensing, biological sensor

## Abstract

Numerous optodes, with fluorophores as the chemical sensing element and optical fibres for light delivery and collection, have been fabricated for minimally invasive endoscopic measurements of key physiological parameters such as pH. These flexible miniaturised optodes have typically attempted to maximize signal-to-noise through the application of high concentrations of fluorophores. We show that high-density attachment of carboxyfluorescein onto silica microspheres, the sensing elements, results in fluorescence energy transfer, manifesting as reduced fluorescence intensity and lifetime in addition to spectral changes. We demonstrate that the change in fluorescence intensity of carboxyfluorescein with pH in this “high-density” regime is opposite to that normally observed, with complex variations in fluorescent lifetime across the emission spectra of coupled fluorophores. Improved understanding of such highly loaded sensor beads is important because it leads to large increases in photostability and will aid the development of compact fibre probes, suitable for clinical applications. The time-resolved spectral measurement techniques presented here can be further applied to similar studies of other optodes.

## 1. Introduction

Sensing of physiological parameters such as pH [1,2,3,4,5,6,7,8], oxygen [2,3,8,9,10,11], glucose [12,13,14], and lactate [15] using fluorescence spectroscopy offers a sensitive technique that has the potential for clinical diagnosis [16,17]. This is achieved by measurement of the fluorescence emission from an analyte-sensitive fluorophore, often with comparison to an insensitive reference reporter. However, the fluorescent emission spectra can be highly dependent on the environment; for instance, it can be affected by pH, polarity, temperature, and proximity to other fluorophores or quenching agents. For in vivo applications, optical fibre sensor systems (optodes) are desirable because they can be placed into remote areas of the human body [18]. The approaches reported include fibre intrinsic interferometers [5], a variety of responsive coatings [2,3,6], and nano or microparticles attached onto the end of the optical fibre [4,8,19,20]. In the latter cases, these sensors often require a high loading density to provide sufficient signal and sensitivities, which we show here may lead to interesting quenching dynamics [21,22,23]. Recently, we demonstrated a new architecture for fibre-based in situ pH and oxygen ratiometric sensing [8], with the sensors based on amino-modified silica microspheres covalently conjugated to fluorophores placed into pits etched on the ends of multi-core optical fibres. Using this approach, we reported pH and oxygen sensing in an ex vivo lung perfusion model with an accuracy of 0.02 pH units and 0.6 mg/L dissolved oxygen sensitivity [8]. Interestingly the optical properties displayed by the silica microspheres with high fluorophore loading densities were intriguing and lead to the detailed studies reported here, which are pertinent to all optical sensing platforms using immobilised fluorescent reporters.

The silica microspheres were loaded with the fluorophore carboxyfluorescein to form so-called “sensor beads”. These were assembled into the etched cores of an optical fibre building the full sensing optode. The use of immobilised carboxyfluorescein (FAM) as a pH indicator in chemical sensors is common, however, while fluorescein and its derivatives exhibit high quantum yields and show pH sensitivity, they are also prone to self-quenching which affects their fluorescence emission [24]. Thus, we reported that high loading density gave an unusual fluorophore response, notably an inverse response to pH (compared to previous reports [24]), but improved sensor stability [8]. Here we studied these highly loaded fluorophore sensors using time-resolved single photon detection methods to explicitly observe the effects of different loadings.

Fibre-based steady-state fluorescence intensity measurements can struggle to separate effects of concentration-dependent signals, photobleaching, and fluorescence from both the optical fibre and the biological samples. Time-resolved fluorescence spectroscopy (TRFS) and fluorescence lifetime imaging microscopy (FLIM) offer the potential to overcome most of these issues [25]. Fluorescence lifetime offers contrast between fluorophores, is not typically affected by photobleaching, and is independent of the concentration of fluorophores (with the exception of at high packing density where quenching effects between the individual fluorophores are no longer negligible as shown here). Beyond this, fluorescence lifetime measurement can provide insight into the relaxation dynamics in excited systems as effected by the fluorophore environment.

Several methods of fluorescence lifetime spectroscopy exist, and are differentiated into time-domain techniques, such as time-correlated single photon counting (TCSPC), and frequency-domain techniques, such as phase fluoroscopy. Here we applied TRFS with TCSPC to study the spectral and time-resolved response of our pH-sensing optode with sensor beads deposited on each multimode core of the multicore fibre. We sampled all 19 cores serially in an automated process to build one measurement set, varying fluorophore loading or external conditions by simply placing the optode into differing buffers prior to measurements. A steady-state commercial spectrometer in combination with a custom-built time-resolved spectrometer recorded the multi-dimensional fluorophore emission characteristics. The intensity, spectral, and time-resolved signals in combination provide insight into the dynamic fluorophore emission processes of such highly loaded beads. Specifically, the advantage of the TRFS with TCSPC study presented here is the ability to investigate such multi-dimensional data, beyond that normally observed in optode development. The study results and techniques used here are relevant to the understanding of any sensors based on high fluorophore loading—particularly relevant for compact optical fibre sensors in which limited space is likely to lead to high fluorophore loading regimes.

## 2. Materials and Methods

### 2.1. Fluorescent Silica Microspheres as Fibre-Based pH Sensors

**Figure 1:**
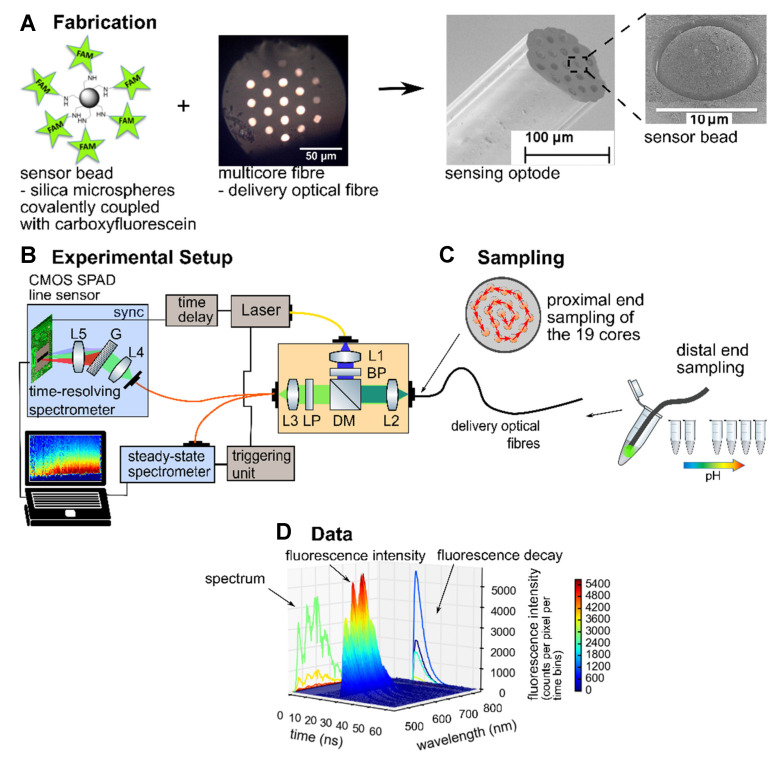
The sensing optode fabrication, experimental setup, and sampling for the time-resolved and steady-state pH measurements: (**A**) the aminomethyl silica microspheres (10 μm, PSI-10.0NH2, Kisker Biotech GmbH & Co., Steinfurt, Germany) were covalently coupled to carboxyfluorescein before being deposited into the etched cores of a 19-core multicore fibre (MCF). Scanning electron microscope (SEM) images of the sensing optode and a bead (functionalised microsphere) are shown. (**B**) Experimental setup used for the investigation of the response of the fluorescence emission to bead loading density and pH changes. The setup comprised a pulsed laser source, a coupling and collection system, and either a steady state spectrometer or a time-resolved spectrometer (Key: L—lens, BP—bandpass filter, DM—dichroic mirror, G—transmission grating, LP—longpass filter). (**C**) The distal end sampling of the fluorescence emission was achieved by inserting the sensing optode into buffer solutions at various pHs and automatically scanning across each of the 19 cores at the proximal end. (**D**) Two-dimensional data of spectrally and time-resolved fluorescence emission from the excited fluorophores.

The protocol for fabrication of the fibre-based pH sensing optode was adapted from Choudhary et al. [8] and is shown in Figure 1.

The aminomethyl silica microspheres (10 μm, PSI-10.0NH2, Kisker Biotech GmbH & Co., Steinfurt, Germany) were covalently coupled to 5(6)-carboxyfluorescein before being deposited into the etched cores of a 19-core multimode multicore fibre (MCF).

Variation in fluorophore density was achieved during coupling chemistry. As previously described [8], the silica microspheres (10 mg, 0.2 mL) were dried and treated with a dimethylformamide (DMF, 0.17 mL) solution of carboxyfluorescein (4.56 mg, 0.012 mmol) pre-activated using Oxima (1.68 mg, 1 eq) and *N*,*N*-diisopropylcarbodiimide (DIC, 1.8 μL, 1 eq), then stirred for 2 h at 50 °C. The carboxyfluorescein solution was diluted with DMF to five different concentrations: Si@FAM-A (no dilution), Si@FAM-B (2 × dilution), Si@FAM-C (6 × dilution), Si@FAM-D (8 × dilution), and Si@FAM-E (10 × dilution) with the reaction volume kept constant. Excess reagents were removed via centrifugation and successive washing with DMF, 20% piperidine/DMF, DMF, methanol and diethyl ether, followed by drying at room temperature.

The silica microspheres were forced into the etched cores by pushing the fibre tip into the microsphere powder such that individual microspheres were forced into the individual cores. Loose microspheres were removed by rinsing thoroughly and wiping with tissue. The microspheres once settled into an etched core are spheres in approximately parabolic pits, and as such were seated firmly. Repeated rubbing on tissue or immersion into the pH buffer had no effect on dislodging the beads. Further assessment of loading repeatability and stability was performed in Choudhary et al. [8], while the later results confirm a reliable response between fibre cores.

### 2.2. Experimental Setup

The fluorescence characteristics—intensity, spectral line shape, and lifetime—of the pH-sensing beads were investigated with a steady-state and a time-resolving spectroscopy system (see Figure 1B). Although the light source and the optical system for coupling and collection were identical, the spectrometers differed. A 485 nm pulsed laser diode (LDH-D-C-485 with PDL 800-D driver, PicoQuant, Berlin, Germany) capable of pulsed or continuous wave (CW) operation was used for excitation and coupled into the coupling-and-collection optical system based around a dichroic beam splitter (Thorlabs, Ely, UK). For the steady state setup, the laser was operated in CW mode, triggered to operate for 100 ms at 10 μW, and the spectra recorded with a simultaneously triggered commercial spectrometer (QE-Pro VIS, Ocean Optics now Ocean Insight, Largo, FL, USA).

For time-resolved measurements the laser was operated in pulsed mode at 20 MHz repetition rate. The time-resolved spectrometer was based on a 256 × 1 pixels complementary metal–oxide–semiconductor (CMOS) single-photon avalanche diode (SPAD) line sensor which allows fast histogramming of arriving photons with time-correlation single photon counting (TCSPC) triggered by the laser source [20,26,27]. The TCSPC capable CMOS SPAD line sensor detects single photons and generates histograms according to their arrival time for 256 pixels simultaneously, each correlated to a different wavelength. An average power of 2 μW was used with an integration time of 10 s to ensure a single-photon regime, avoiding pile-up effects, and recording sufficient signal (photons) for a quantitative analysis.

The measurements were taken in both modalities (steady state and time-resolved fluorescence) with the fibre dipped into a phosphate buffer system at various pHs (5.8, 6.2, 6.5, 7.2, 7.8, 8.1, 8.5) in a randomised order, as indicated in Figure 1C. The pH buffers were checked before measurements with a commercial pH meter (Mettler Toledo, Columbus, OH, USA). For each measurement the sensing optode was placed into the pH buffer for 30 s to stabilise before measurement. Light was coupled consecutively into each of the 19 cores automatically (see Figure 1C) via a motorized x–y–z stage (Nanomax, Thorlabs, Ely, UK). For the steady state experiment the triggered 100 ms measurements were performed automatically at each core. The time-resolved measurement was manually initiated. Three complete steady-state measurement series (of 7 randomised pH buffers) were performed, followed by a time-resolved measurement series, then a final, fourth, steady-state measurement series.

### 2.3. Data Analysis

For the steady-state measurement, the spectra were background subtracted via the OceanView software (OceanOptics, now Ocean Insight, Largo, FL, USA). Empty cores were omitted, as were cores with extremely weak signals indicating the microsphere was not well seated. The remaining spectra were compared by evaluating the integrated fluorescence intensity and the intensity-weighted mean wavelength. The variance between cores provided the statistics shown in later figures.

Figure 1D shows an example of the two-dimensional data of the spectrally and time-resolved fluorescence emission from an excited fluorophore. To derive the fluorescence lifetimes from the measured decay curves, a non-linear curve fitting method was used and optimised to reduce the chi-squared error. To avoid distortion at the beginning of the decay, a tail fit was performed omitting the first time bin. The instrument’s response function was not deconvolved, resulting in slightly increased derived lifetimes (this does not affect the conclusions because all of the compared lifetimes would be affected in the same way). The more diluted fluorophores fitted well with a single exponential function whereas the more densely loaded beads (Si@FAM-A and Si@FAM-B) were found to fit best with a double exponential function and a single lifetime derived as an amplitude-weighted function of both fluorescence lifetimes [23,28].

## 3. Results and Discussion

### 3.1. Changes in the Fluorescence Emission with Bead Loading Density

**Figure 2:**
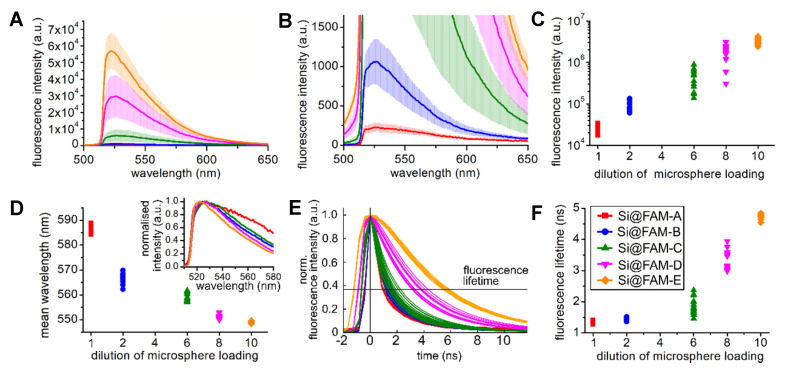
Changes in the fluorescence emission with varying fluorophore loadings on the beads. The loading solution of Si@FAM-E was 10× diluted compared to that of Si@FAM-A. All measurements aretaken at pH 7.2. The legend for all figures is placed in (**F**): (**A**) The fluorescence spectra. The line is the mean of the intensities from the cores, the standard deviation is shown as shaded area. (**B)** For visibility, the same data is expanded to illustrate the lower fluorescence intensity signals. (**C**–**F**) show the data from all beads on each plot. (**C**) Fluorescence intensity (log scale) calculated as the area under the curve of the fluorescence spectra. (**D**) Mean wavelength of the fluorescence spectra line shape (inset shows the normalised mean spectra). (**E**) Normalised fluorescence decays from the data summed across the 520 to 700 nm spectral range. (**F**) Fluorescence lifetime, derived from the decays in (**E**) via an exponential fitting routine.

Figure 2 shows the variation of the fluorescent emission in intensity, line shape, and fluorescence lifetime with different fluorophore loadings on the beads (all measurements are taken at pH 7.2).

Labelling references the dilution of the fluorophore during loading onto the sensors as described in Section 2.1, such that Si@FAM-E is a substantially lower fluorophore density than Si@FAM-A. To quantify changes in fluorescence response observed in Figure 2A,B, the integrated intensities and the mean wavelengths are plotted versus the loading dilution for all cores in Figure 2C,D, respectively. Fluorescence intensity significantly decreased with increasing bead loading (moving left on the axis), and the spectral line shapes were red-shifted and broadened [24]. It was apparent that intermediate bead loadings (Si@FAM-C and Si@FAM-D) exhibited the greatest fluctuations in intensity between cores.

The calculated mean wavelength quantifies changes in the spectral line shape of the whole optode. This includes the red-shift and broadening, but also an increasing protrusion from the fluorescent background of the fibre at longer wavelength when fluorescence signals from the beads are of lower intensity, e.g., Si@FAM-A and Si@FAM-B. This will influence the trend shown in Figure 2D. However, as can be seen, broadening of the peak of the bead fluorescence is clear. 

In Figure 2E the normalised fluorescence decays for each bead loading are displayed and Figure 2F shows the fitted fluorescence lifetimes across all cores. A significant decrease in fluorescence lifetime for beads with greater loading can be seen. Additionally, we observe a shorter rise time for these beads.

The observed features of decreasing fluorescence intensity, spectral broadening, and decreasing fluorescence lifetime with increasing bead loading indicate a quenching effect between the fluorescent molecules. On the highly loaded beads (e.g., Si@FAM-A), the fluorescent molecules are in such close proximity that intermolecular self-quenching occurs. According to the manufacturer, the microspheres have 1 × 10^9^ free NH units on their surface to which fluorophore molecules can attach.

If fully occupied, the spacing between molecules is ~0.5 nm. At this spacing additional depopulation paths of the fluorescence states occur, such as intersystem crossing or Förster resonance energy transfer (FRET), resulting in fluorophore quenching in which carboxyfluorescein is both donor and acceptor. To be explicit, the fluorophores in close proximity form a coupled excitation energy level system with increased degeneracy. This results in increased decay routes after photon absorption which can allow the excited fluorophore to silently relax (with reduced photon emission). Similarly, due to the degeneracy of relaxation mechanisms, this occurs on a short timescale, thus exhibiting decreased fluorescence lifetime. Of the emissive decay routes, there are more available with lower energy separation, resulting in spectral broadening (red-shifts). The observed dependencies in Figure 2 correlate well with the expected increased coupling between fluorophores as bead loading increased. The most explicit observation is the fluorescent lifetime, reducing from values expected for fluorescein derivatives at low loading (Si@FAM-E), to a short lifetime below the resolution of our system (likely well below 1 ns), indicating a heavily coupled or quenched system (Si@FAM-A). It is interesting to note the prolonged rise time and the flattened peak in Figure 2E for the less quenched fluorophores. This is not included in the definition of the fluorescence lifetime and therefore the fit to the decay, but indicates greater change in the dynamics than we enumerate in Figure 2F.

Some variation in fluorophore signal strength is expected from bead size variations, and therefore seating, of the microspheres in the pits of the fibre. However, it is notable that in Figure 2C the intermediate loading densities (Si@FAM-C and Si@FAM-D) have greater amplitude fluctuations between cores; this is also observed in Figure 2E,F for the lifetime. This represents the fluorophore being partially quenched, and therefore in a more unstable state sensitive to slight fluctuations in surroundings.

### 3.2. Photostability

**Figure 3:**
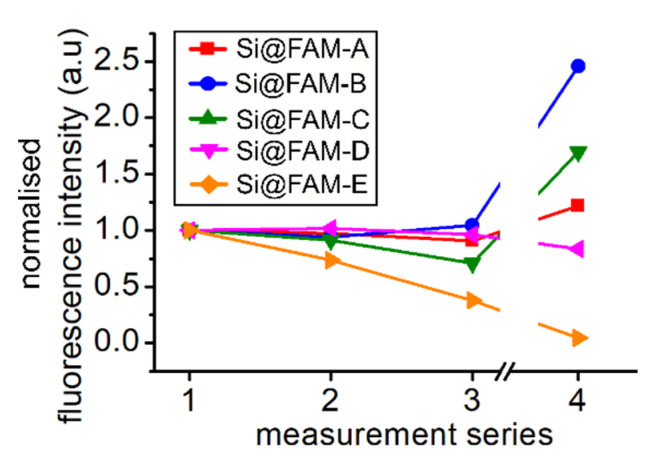
Sensor photostability at different bead loadings observed as changes in the fluorescent intensity over repeated measurements. All measurements were taken at pH 7.2.

Fluorescein and its derivatives are affected by photobleaching, which limits their utility for repeated measurements or monitoring in vivo. To assess the photostability of the quenched fluorophores, the averaged fluorescence intensities over all cores for pH 7.2 between four intensity-based measurement series are shown in Figure 3.

Importantly, the time-resolved measurements were performed between the third and fourth series with an average power of 2 μW at 20 MHz repetition rate with pulse widths of <100 ps, which leads to a peak power of ~1 mW during each pulse. Photobleaching is a non-linear effect, where a multiply excited fluorophore becomes permanently changed. As such, even with lower average powers, short higher peak power pulses have a much stronger photobleaching effect than constant low power illumination. In addition, the time-resolved measurement took place over 10 s for each core, resulting in notable potential for photobleaching compared to the steady state measurements (100 ms duration per core, 10 μW).

In Figure 3 we observe that the majority of beads showed a reasonably stable fluorescence during the three repeats of steady state measurements, with only more notable changes observed in the fourth measurement. However, the greatest variation is visible in the less densely loaded beads, Si@FAM-E, which shows significant photobleaching over the measurements. This is in direct agreement with results presented in Choudhary et al. [8] (Supplementary Information), in which signal degradation is examined during repeated illumination.

An interesting response is observed in Figure 3 for the fourth measurement set (after more aggressive pulsed illumination). Here the less densely loaded Si@FAM-E exhibited notable bleaching as might be expected, but upon moving to higher loadings the intensity is observed to increase after intense illumination. This is perhaps most notable for intermediate loading regimes (e.g., Si@FAM-B and Si@FAM-C).

Limited bleaching under normal illumination is consistent with more numerous relaxation paths present for the quenched fluorescence molecules. The increased signal after intense illumination shows that the destruction of some fluorophores results in an effective reduction of the loading density and hence the quenching effect. The outcome is an increase in fluorescence intensity.

### 3.3. Response to pH

**Figure 4:**
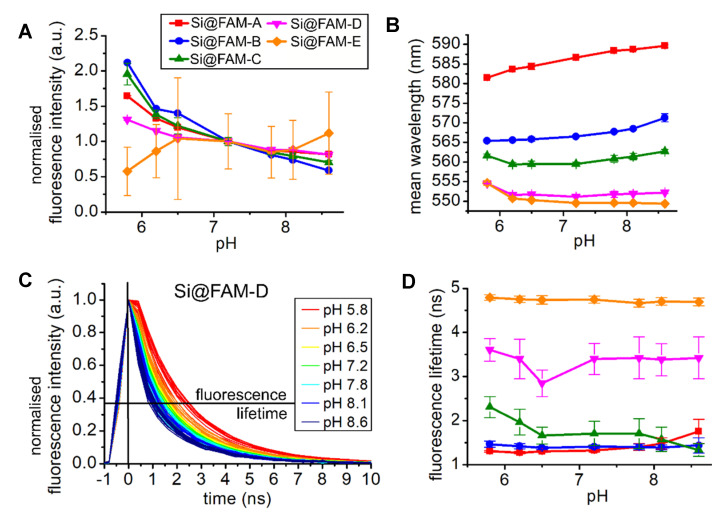
Response to pH of beads with varying loading density. Three series of measurements were taken over a range of physiological relevant pH from 5.8 to 8.6. The legend for (B), and (D) is placed in (**A**): (**A**) Changes in the fluorescence intensity with pH; displayed are the mean and its standard deviation across all cores. For reason of comparison, all fluorescence intensities are normalised to their value at pH 7.2. (**B**) Mean wavelength of the fluorescence spectra line shape with pH. (**C**) Normalised fluorescence decays of beads Si@FAM-C showing a decrease in lifetime with increasing pH. The spectral histograms are summed over the range 520 to 700 nm for each core, and the results for 10 cores at each pH are shown. (**D**) Fluorescence lifetime changes with pH for various bead loading densities.

The pH sensitivity of the functionalised microspheres at various bead loadings was accessed in pH buffers in a physiologically relevant range (pH 5.8 to 8.6) in randomised order over three repetitions. Carboxyfluorescein is a known pH sensitive molecule and the expected behaviour is that the fluorescence intensity increases with increasing pH [24]. Figure 4A compares the response to pH by normalizing the fluorescence intensity changes to the value at pH 7.2.

The experiments were all performed at room temperature. However, the responses to pH of fluorescein may be expected to vary with temperature, and as such calibration would instead be performed at ~37 °C for anticipated in vivo use to match the core body temperature.

The less densely loaded Si@FAM-E beads displayed a factor of 2 change in intensity across the range (matching that expected from the literature [24]) decreasing with lower pH. However, large error bars show the variation between cores and across measurement repetitions. As the pH buffer order was randomised, and notable photobleaching has been observed for this sensor (Figure 3), the large variability in response is expected. The more highly loaded beads exhibited a very different response, inverting the usual response with increasing fluorescence intensity at lower pH, as first observed in Choudhary et al. [8]. The previously observed stability of these beads (Figure 3) in combination with repeatability between the cores results in relatively small error bars and a reliable pH sensor.

Changes in the spectral line shape due to pH responses were quantified with the mean wavelength (see Figure 4B). The higher loaded beads showed a red-shift compared to those with a lower loading, increasing at higher pH (most apparent for Si@FAM-A and Si@FAM-B). The beads with the highest loading exhibited a fluorescent lifetime below that of our measurement system (time resolution ~400 ps and Instrument Response Function (IRF) ~1 ns [1,2]. Lower loaded beads exhibited the expected longer lifetimes, however, Figure 4C,D highlight that the fluorescence lifetime of an intermediate fluorophore bead loading changes in an interesting way in response to pH. A decrease in lifetime is observed at higher pH, where normally an increased intensity signal would also be expected, but here a decrease is seen. The explicit observation of decreasing lifetime at higher pH is consistent with increased quenching occurring, reducing both emission intensity and lifetime when an isolated fluorophore would have been more active. This is also consistent with the observed increased red-shift (spectral broadening) seen at higher pH. 

### 3.4. Ratiometric Dual Fluorophore Optode

**Figure 5:**
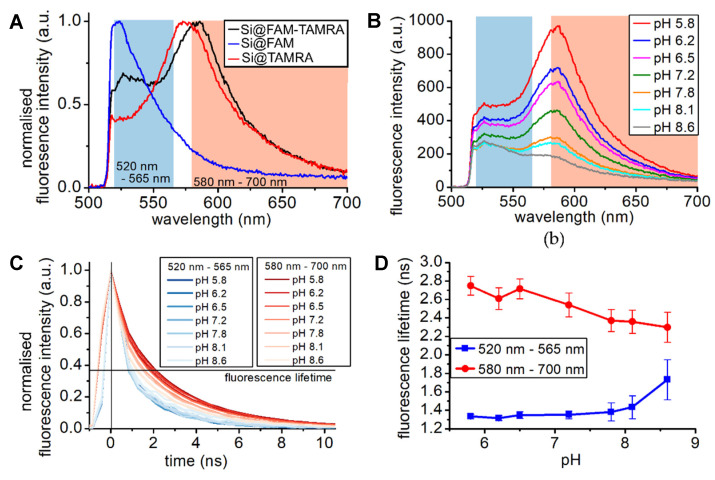
Response to pH of a dual fluorophore optode: (**A**) Fluorescence spectra of the dual fluorophore loaded beads, Si@FAM-TAMRA, and beads loaded only with carboxyfluorescein (Si@FAM) and tetramethylrhodamine (Si@TAMRA). The shaded area indicates the chosen carboxyfluorescein dominated spectral region (blue) and the tetramethylrhodamine dominated spectral region (red). (**B**) Spectral changes of the ratiometric sensor with pH. (**C**) Normalised fluorescence decays; the spectral histograms were summed over the range of 520 to 565 nm for the carboxyfluorescein dominated spectral region (blue) and from 580 to 700 nm for the tetramethylrhodamine spectral region (red). (**D**) Fluorescence lifetime, derived from the fluorescence decays via an exponential fitting routine.

The ratiometric dual fluorophore optode was developed in the same way as described for the carboxyfluorescein optode with the silica beads loaded with two fluorophores (carboxyfluorescein (FAM) and tetramethylrhodamine (TAMRA) in a ratio of 300:1). The carboxyfluorescein loading corresponds to that of Si@FAM-A [8]. Although the fluorescence intensity of carboxyfluorescein changes with pH, tetramethylrhodamine does not and could thus be used as an in-measurement reference point to improve pH measurements. However, in a highly coupled system the situation is more complex. Figure 5 investigates the changes in fluorescence spectra and lifetime of the dual fluorophore loaded beads, Si@FAM-TAMRA, to investigate the coupling.

Further calibration of signal amplitude response to pH is provided in Choudhary et al. [8]. Figure 5A compares the fluorescence spectra of the dual fluorophore beads with those that were loaded individually with carboxyfluorescein, Si@FAM, and tetramethylrhodamine, Si@TAMRA. The spectral regions where each fluorophore is dominant (chosen for the greatest contrast in lifetime) are highlighted and were used for the time-resolved analysis.

Figure 5B shows the spectral changes of the combined fluorescence system in response to pH. The intensities of the characteristic tetramethylrhodamine emissions changed strongly, and the carboxyfluorescein region relatively little. Figure 5C shows the pH dependency of the normalised fluorescence decays, and Figure 5D shows the fitted fluorescence lifetimes, from the carboxyfluorescein (blue) and tetramethylrhodamine (red) dominated regions of the spectra. The carboxyfluorescein dominated decays show a short fluorescence lifetime similar to that seen in the highly loaded carboxyfluorescein beads, e.g., Figure 2E Si@FAM-A. The tetramethylrhodamine dominated region shows a longer lifetime for tetramethylrhodamine (expected fluorescence lifetime of ~2.3 ns), which decreases with increasing pH. Again, we noticed the further difference in the rising dynamics of the fluorescence decay for the carboxyfluorescein and tetramethylrhodamine dominated regions, not represented in the fitted lifetime.

These results describe a classic FRET pair where carboxyfluorescein is donor and tetramethylrhodamine acceptor, loaded at high density and therefore exhibiting a joint response to environmental changes [21,23,29]. As previously discussed for the case of highly coupled carboxyfluorescein systems, the response here describes energy transfer and quenching occurring. The carboxyfluorescein changes with pH are being transferred to the tetramethylrhodamine intensity responses [30,31]. At high pH, the fluorescein more actively quenches the tetramethylrhodamine, exhibiting reduced intensity and lifetime at the longer wavelength region. Furthermore, the coupled system also pulls up the lifetime of the carboxyfluorescein dominated region. Explicit combined spectral and lifetime evaluation offers insight into such FRET pairs and could be used for the study of other complex systems.

## 4. Conclusions

Fibre-based endoscopic fluorescence sensors have the potential for clinical in vivo application to enhance the understanding of physiological parameters in disease pathology. The need for miniaturised optodes introduces a requirement for high fluorophore density due to the small practical volume. An example of this, based on silica microspheres, was thoroughly characterised here in a spectrally and time-resolved manner. These investigations describe the opportunity for optical fibre time-resolved spectroscopy to provide insight into fluorophore response, especially in the case of densely loaded fluorophores on the ends of optical fibres. However, these techniques are also applicable to investigation of spectral fluorescence dynamics of other samples, such as endogenous tissues.

Here fluorophores were attached covalently to amino-modified silica microspheres that were seated into the etched cores of a 19 core MCF. For five fibres with beads with differing loadings, all cores were characterised utilising an automated alignment system to provide ensemble measurements. We observed decreasing fluorescence intensity, spectral red-shift, and decreasing fluorescence lifetime with increasing bead loading density, which explicitly demonstrates the quenching effect due to energy transfer between the fluorescent molecules. The most densely loaded beads exhibited stable fluorescence across multiple measurement series in comparison to those with reduced loading densities, indicating a limited photobleaching effect under normal illumination as numerous relaxation paths are present for the quenched fluorescence molecules. The fluorescence pH response of these microsensors was evaluated in the physiologically relevant region from pH 5.8 to 8.6. The most densely loaded beads showed a complex quenching effect which reversed the normal influence of pH on fluorescein intensity, decreasing intensity and lifetime with increasing pH.

Finally, we investigated a ratiometric microsensor loaded with carboxyfluorescein and tetramethylrhodamine, which also exhibited strong energy transfer between the fluorophores of a classic FRET pair. Here the observed response to pH shows decreasing tetramethylrhodamine fluorescence intensity with increasing pH, and a converging fluorescence lifetime of both fluorophores with increasing pH due to the coupling between the molecules. The time-resolved features attributable to the distinct fluorophores are separable through the spectral capabilities of our detection system.

Miniaturised fibre-optic based sensors for physiological parameters could have the potential to aid clinical diagnostics in the future, providing key information about physiological parameters. However, a high loading density of the reporter fluorophore is desirable to achieve sufficient sensitivity. This leads to altered fluorescence emission and dynamics that can be traced back to quenching effects. Our results indicate that the sensors work better in these highly loaded regimes and the findings presented in this work are applicable to the development, design, and engineering of next generation fibre-optic biosensors [7,8,32,33] for in vivo analysis of physiological parameters.

## Supplementary Materials

The data presented in this study are openly available on Edinburgh DataShare at [https://doi.org/10.7488/ds/2934].
